# Isolated Third Nerve Palsy as a Rare and Solo Presentation of Internal Carotid Artery Dissection in a Young Female - a Surprising Finding in the Angiogram

**DOI:** 10.7759/cureus.14035

**Published:** 2021-03-22

**Authors:** Randa Abdelmasih, Ramy Abdelmaseih, Elio Monsour, Justin Reed

**Affiliations:** 1 Internal Medicine, University of Central Florida College of Medicine, Ocala, USA

**Keywords:** internuclear ophthalmoplegia, third nerve palsy, internal carotid artery aneurysm, angiogram

## Abstract

Internal carotid artery dissection (ICAD) is a known but uncommon cause of ischemic stroke among young and middle-aged patients. A common presentation includes ipsilateral headache, unilateral oculosympathetic palsy (partial Horner syndrome), or ischemic stroke but some reported cases present with less common manifestations, such as lower cranial nerve syndrome (IX, X, XI, XII). However, third cranial nerve palsy is an extremely rare presentation of ICAD. We present a case of ICAD with pseudoaneurysm presenting with third nerve palsy, with ptosis, outward deviation, and binocular diplopia, emphasizing the importance of considering ICAD as a differential diagnosis in patients with third nerve palsy due to the anatomical proximity of ICA to third nerve within the cavernous sinus.

## Introduction

Internal carotid artery dissection (ICAD) is a known but uncommon cause of ischemic stroke among young and middle-aged patients, accounting for 14-20% of all cases [1,2]. ICAD is a sudden intimal tear in the ICA that occurs usually secondary to trauma or connective tissue disorders but many reported cases appear spontaneous. This tear allows blood to flow into the intimal layer of the vessel forming a hematoma. A thrombus may then form, leading to stenosis of the lumen and dilatation of the artery or pseudoaneurysm formation. Common presentations include ipsilateral headache, unilateral oculosympathetic palsy (partial Horner syndrome), or ischemic stroke but some reported cases present with less common manifestations such as lower cranial nerve syndrome (IX, X, XI, XII). However, third cranial nerve palsy is an extremely rare presentation of ICAD. Though, the mechanism of this association is not quite clear. 

We present a case of ICAD with pseudoaneurysm presenting with third nerve palsy, emphasizing the importance of considering ICAD as a differential diagnosis in patients with third nerve palsy due to the anatomical proximity of ICA to third nerve within the cavernous sinus.

## Case presentation

A 32-year-old female with a past medical history of Hashimoto's thyroiditis and migraine presented with pressure-like left retro-orbital eye pain associated with double vision, nausea, and emesis beginning approximately three days prior. Since then, her pain intensified, prompting her to see her primary care provider who diagnosed an eye vs. sinus infection and prescribed an antibiotic, nasal and oral steroid, which failed to improve her symptoms. Of note, she had experienced a double vision in the same eye one year prior and was evaluated by a neuro-ophthalmologist who told her this was due to ocular muscle weakness and prescribed her prescription lenses. 

**Figure 1:**
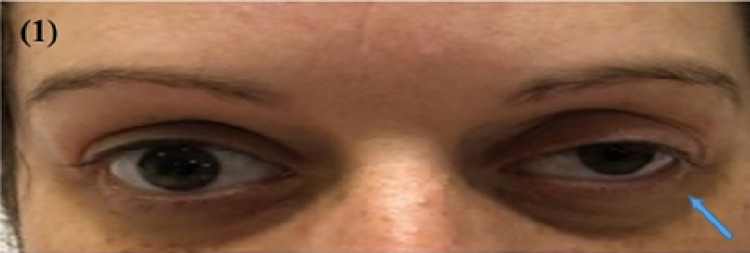
Eye examination: arrow pointing at left partial ptosis and outward deviation of the left eye

**Figure 2:**
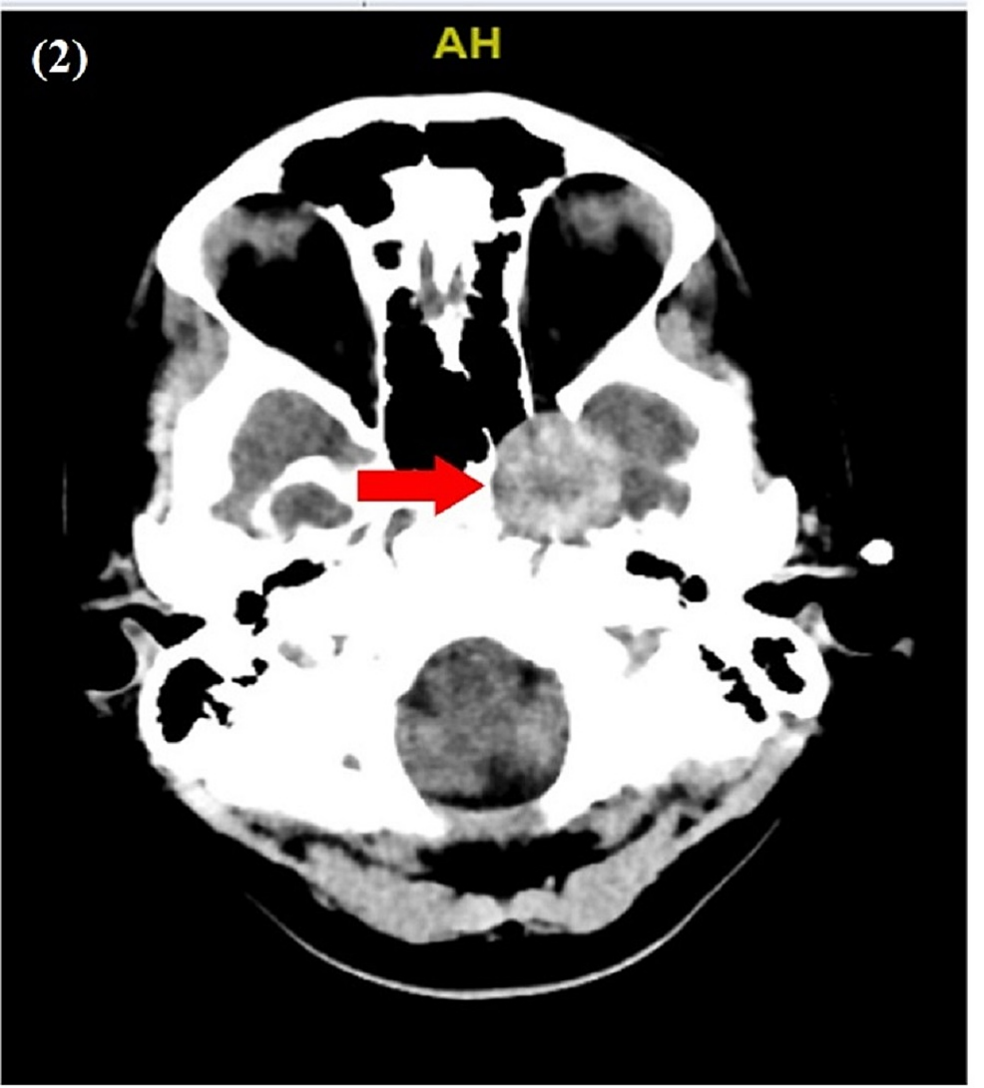
CT brain without contrast: arrow showing a mixed-density mass measuring 2.7 x 2.6 cm, which is seen in the medial aspect of the left middle cranial fossa adjacent to the left temporal lobe suggesting an intracranial neoplasm

**Figure 3:**
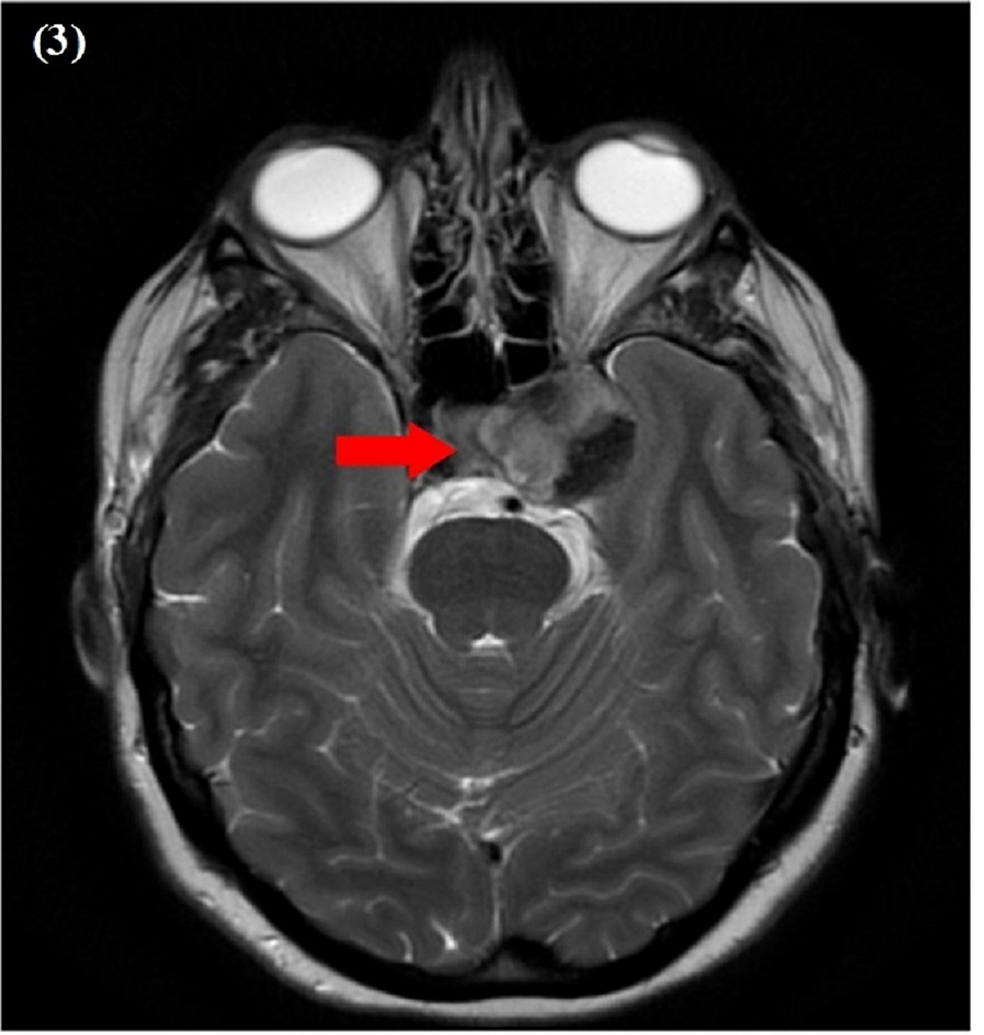
MRI brain with and without contrast: arrow showing an extra-axial tumor, measuring 3.3 x 2.4 x 3.0 cm, at the level of the left cavernous sinus with evidence of concavity involving the pituitary gland

**Figure 4:**
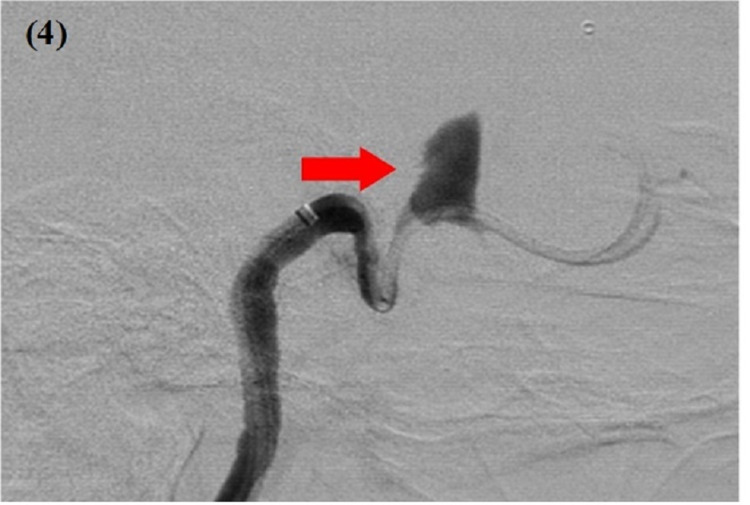
Diagnostic cerebral angiogram: arrow demonstrating a partially thrombosed left cavernous internal carotid artery pseudoaneurysm

On admission, she was hemodynamically stable and afebrile. Physical examination was remarkable for tenderness to palpation around the left eye. A neurological examination of the eye was consistent with left third nerve palsy. By inspection, there was left eye partial ptosis with outward deviation and dilated pupil, which responded poorly to light. Additionally, paralysis of left eye adduction and elevation with binocular diplopia maximized by looking to the right was noted. Other cranial nerves were normal (Figure 1). Labs were only remarkable for a white blood cwll count of 13.7. Computed tomography (CT) brain without contrast revealed a mixed-density mass measuring 2.7 x 2.6 cm, seen in the medial aspect of the left middle cranial fossa adjacent to the left temporal lobe (Figure 2). Magnetic resonance imaging (MRI) brain with/without contrast showed an extra-axial tumor measuring 3.3 x 2.4 x 3.0 cm at the level of the left cavernous sinus with evidence of concavity involving the pituitary gland (Figure 3). Differential diagnosis per radiology team was meningioma, melanocytoma, and nerve sheath tumor. The patient was started on intravenous methylprednisone. Neurosurgery was consulted and an angiogram was considered to manage accordingly. The patient underwent a cerebral angiogram, which showed grade III dissection of the left cavernous ICA with a large partially thrombosed pseudoaneurysm (Figure 4). The patient was then loaded with clopidogrel and aspirin and then she underwent an angiogram for a left cavernous ICA flow diversion stent embolization procedure. She was discharged on clopidogrel, aspirin, and steroid tapering dose to follow up with neurosurgeon outpatient.

## Discussion

Oculomotor nerve palsy secondary to ICAD is extremely rare but has been reported. Though the mechanism of this association is not quite clear, it can be explained by hypoperfusion of the arteries feeding the third cranial nerve secondary to dissection-induced low flow, microembolism from thrombosed pseudoaneurysm, or nerve compression [3]. 

Our patient’s presentation was consistent with left third nerve palsy with ptosis, outward deviation, and binocular diplopia maximal when looking to the right. Initial imaging including CT and MRI was consistent with intracranial mass with differential diagnosis of pituitary adenoma, meningioma, and nerve sheath tumor. As our patient was young with no significant past medical history, cervicocerebral dissection was considered as a differential, though it was not manifested on brain CT and MRI [4]. Thus, the decision of angiogram was taken before the further intervention, which subsequently revealed the diagnosis. Currently, there is no class I guidelines to direct choice of therapy. Available treatments consist of antithrombotics including anticoagulation versus antiplatelet agents. Interventional options for treatment include endovascular coiling, flow diversion stent, or catheter-directed thrombolysis. 

## Conclusions

Although rare, cranial nerve palsy can be the only manifestation of ICAD and should be considered especially if the patient is young and has no other risk factors.
